# Supplementary material for: Smear positive tuberculosis and genetic diversity of M. tuberculosis isolates in individuals visiting health facilities in South Gondar Zone, northwest Ethiopia
Source: PLoS One. 2019 Aug 8;14(8):e0216437. doi: 10.1371/journal.pone.0216437 (PMC6687116; doi:10.1371/journal.pone.0216437)
Supplement: S1 Appendix — The consent form was designed for the study participants or witnesses in case of illiterate ones who attend health facilities during the study period in South Gondar Zone, northwest Ethiopia. (PDF) [file pone.0216437.s001.pdf]

S1 Appendix

Informed consent form for individuals who attend health centers/hospital in South Gondar Zone of the Amhara Region, and who are inviting to participate in research on tuberculosis. The title of the research project is **"Smear positive tuberculosis and genetic diversity of *M. tuberculosis* isolatesin individuals visiting health facilities in South Gondar Zone, northwest Ethiopia''**

This Informed Consent Form has two parts:

- **Information Sheet (to share information about the research with the attendants)**
- **Certificate of Consent (for signatures if the attendants agree to take part in the research)**

The attendants will be given a copy of the full Informed Consent Form

PART I: Information Sheet

Introduction

We are doing research on human tuberculosis disease, which is very common in this country. I am going to give you information and invite you to be part of this research. You do not have to decide today whether or not you will participate in the research. Before you decide, you can talk to anyone you feel comfortable with about the research.

There may be some words that you do not understand. Please ask me to stop as we go through the information and I will take time to explain. If you have questions later, you can ask them of me, the study doctor or the staff.)

Purpose of the research

Globally, tuberculosis (TB) causes millions of deaths per year. Ethiopia ranks seventh among the worldôs 22 countries with high tuberculosis burden. *Mycobacterium tuberculosis(M. tuberculosis)* is the most common cause of human TB. Tuberculosis is one of the most common and dangerous diseases in this region. However, the situation is not well known among farmers. Particularly, in South Gondar Zone, no enough recorded data on the transmission of the disease.

Considering the above facts, it is planned to undertake a research on tuberculosis in the next two years among farmers in south Gondar zone of the Amhara region. The research will be undertaken with coordination involving many professional personnel mainly health experts as well as researchers will participate.

The objectives of the study are:

a) To determine the magnitude in the distribution of tuberculosis in South Gondar Zone

b) To determine the genetic diversity of the *M. tuberculosis* in the study area.

c) To investigate the association of different risk factors with tuberculosis.

With this respect, a research on human tuberculosis will be undertaken at the woreda health centers and fields in south Gondar zone.

Type of Research Intervention

This research will involve collection of a sputum sample (during the first health center visit) or a biopsy at swollen lymph nodes from TB suspected individuals, **only sharing the samples from the routine TB diagnosis at health centers (Samples will be taken only for the purpose of the research)**, an interview as well as a single injection at two sites in the neck region of your cattle at the field.

Participant selection

We are inviting all individuals above five years of age with tuberculosis who attend health centers in South Gondar Zone to participate in the research on cattle and human tuberculosis.

Voluntary Participation

Your participation in this research is entirely voluntary. It is your choice whether to participate or not. Whether you choose to participate or not, all the services you receive at this clinic will continue and nothing will change. If you choose not to participate in this research project, you will be offered the treatment that is routinely offered in this clinic/hospital for TB, and we will tell you more about it later. You may change your mind later and stop participating even if you agreed earlier.

Procedures and Protocol

You will receive the treatment of your condition according to national guidelines. This means that you will be given anti-TB drugs and consultations free of charge from the health center.

Description of the Process

During the research you make only one visit to the health center and we will make two visits to your farm land where your cattle are found.

- During your visit to the health center/hospital in seeking TB treatment, a small amount of sputum, equal to about a teaspoon, will be collected from your mouth with a small container. This sputum will be tested for the presence of bacteria that cause human or cattle tuberculosis. We will also ask you a few questions about your habit of consuming raw milk and diary products, raw meat and your association with cattle at your home or farm, in addition, we will also ask you about your general awareness about human and cattle TB.
- In the presence of swellings as TB indications and to confirm the cause of your swelling, a small sample of your skin will be taken. The guidelines say that the sample must be taken using a local anesthesia which means that we will give you an injection close to the area where we will take the sample from. This will make the area numb so that you will not feel any pain when we take the sample.

Risks

By participating in this research it is possible that you will feel some discomfort and may lose some time in coming to the health center otherwise there will not be given any new medicine or treatment that might harm your health. The fine needle aspirations (FNA) will be carried out by experienced pathologists at hospital level as part of the routine diagnosis using appropriate aseptic (safety) measures. Commonly, you may feel a little sore for a couple of days after the test. You may develop a bruise at the site where the needle was inserted. Otherwise, complications are uncommon. But if it happens, you will get appropriate treatment at the hospital.

Benefits

There may not be any benefit for you but your participation is likely to help us find the answer to the research question. There may not be any benefit to the society at this stage of the research, but future generations are likely to benefit.

Confidentiality

The information that we collect from this research project will be kept confidential. Information about you that will be collected during the research will be put away and no-one but the researchers will be able to see it. Any information about you will have a number on it instead of your name. Only the researchers will know what your number is and we will lock that information up with a lock and key. It will not be shared with or given toanyone except Addis Ababa University and the health officials at the health center. The biological samples obtained during this research procedure will be used only for this research, and will be destroyed after two years, when the research is completed.

Sharing the Results

The knowledge that we get from doing this research will be shared with you through community meetings before it is made widely available to the public. Confidential information will not be shared. There will be small meetings in the community and these will be announced. After these meetings, we will publish the results in order that other interested people may learn from our research.

Who to Contact

If you have any questions you may ask them now or later, even after the study has started. If you wish to ask questions later, you may contact any of the following:

Prof. Gobena Ameni: *Addis Ababa University, Aklilu Lemma Institute of Pthobiology. Office tel.: +251-112 76309, Mobile: +251-911 413073, University email ID: gobena.ameni@aau.edu.et*

Amir Alelign: AddisAbaba University, College of Life Science, Department of Microbial, Cellular and Molecular Biology. Office tel: +251-8959216, Mobile: 0911380705. Email ID: negaalelign@yahoo.com

This proposal has been reviewed and approved by Addis Ababa University, College of Natural Science IRB, which is a committee whose task it is to make sure that research participants are protected from harm.

PART II: Certificate of Consent

I have read the foregoing information, or it has been read to me. I have had the opportunity to ask questions about it and any questions that I have asked have been answered to my satisfaction. I consent voluntarily to participate as a participant in this research.

Name of Participant__________________

Signature of Participant ___________________

Date ___________________________

Day/month/year

If illiterate

A literate witness who is selected by the participant will sign.

I have witnessed the accurate reading of the consent form to the potential participant, and the individual has had the opportunity to ask questions. I confirm that the individual has given consent freely.

Name of witness_____________________ AND Thumb print of participant

Signature of witness ______________________

Date ________________________

Day/month/year

Statement by the researcher/person giving consent

I have accurately read out the information sheet to the potential participant, and to the best of my ability made sure that the participant understands that the following will be done:

1. Sputum samples or a biopsy at swellings will be collected

2. Skin test on cattle will be done at the farm land of the participants

3. TB positive cattle will be sacrificed in agreement with the owners and samples will be taken for further study.

I confirm that the participant was given an opportunity to ask questions about the study, and all the questions asked by the participant have been answered correctly and to the best of my ability. I confirm that the individual has not been coerced into giving consent, and the consent has been given freely and voluntarily.

A copy of this ICF has been provided to the participant.

Name of Researcher/person taking the consent________________________

Signature of Researcher /person taking the consent__________________________

Date ___________________________

Day/month/year
